# Cuticular Hydrocarbon Content that Affects Male Mate Preference of *Drosophila melanogaster* from West Africa

**DOI:** 10.1155/2012/278903

**Published:** 2012-03-28

**Authors:** Aya Takahashi, Nao Fujiwara-Tsujii, Ryohei Yamaoka, Masanobu Itoh, Mamiko Ozaki, Toshiyuki Takano-Shimizu

**Affiliations:** ^1^Department of Population Genetics, National Institute of Genetics, Mishima 411-8540, Japan; ^2^Department of Genetics, The Graduate University for Advanced Studies (SOKENDAI), Mishima 411-8540, Japan; ^3^Science and Technology, Graduate School of Science and Technology, Kyoto Institute of Technology, Kyoto 606-8585, Japan; ^4^Laboratory of Insect Behavior, National Institute of Agrobiological Sciences (NIAS), Ohwashi 1-2, Tsukuba, Ibaraki 305-0851, Japan; ^5^Center for Bioresource Field Science, Kyoto Institute of Technology, Kyoto 606-8585, Japan; ^6^Insect Biomedical Research Center, Kyoto Institute of Technology, Kyoto 606-8585, Japan; ^7^Department of Biology, Faculty of Science, Kobe University, Kobe 657-8501, Japan; ^8^Department of Biological Sciences, Graduate School of Science, The University of Tokyo, Tokyo 113-0033, Japan

## Abstract

Intraspecific variation in mating signals and preferences can be a potential source of incipient speciation. Variable crossability between *Drosophila melanogaster* and *D. simulans* among different strains suggested the abundance of such variations. A particular focus on one combination of *D. melanogaster* strains, TW1(G23) and Mel6(G59), that showed different crossabilities to *D. simulans*, revealed that the mating between females from the former and males from the latter occurs at low frequency. The cuticular hydrocarbon transfer experiment indicated that cuticular hydrocarbons of TW1 females have an inhibitory effect on courtship by Mel6 males. A candidate component, a C25 diene, was inferred from the gas chromatography analyses. The intensity of male refusal of TW1 females was variable among different strains of *D. melanogaster*, which suggested the presence of variation in sensitivity to different chemicals on the cuticle. Such variation could be a potential factor for the establishment of premating isolation under some conditions.

## 1. Introduction

*Drosophila* exhibits complex mating behavior with frequent wing vibration and copulation attempts by males. The successful mating is achieved by communications between males and females using chemical, acoustic, and visual signals (reviewed in [1]). Subtle differences in these signals may accumulate during or after the formation of reproductive isolation. Once reproduction isolation is established to a certain extent, the correct mate recognition is essential to avoid costly hybridization and wasting time on unsuccessful courtship. Indeed, a certain degree of premating isolation or mating incompatibility is commonly observed between closely related species of *Drosophila* [2, 3].

In some cosmopolitan species of *Drosophila*, for example, *D. ananassae* [4] and *D. elegans* [5, 6], widely observed mating incompatibilities between populations from different locations exist. The degree of incompatibility is variable among sampled strains in these species. Another cosmopolitan species, *D. melanogaster*, also harbors incompatible combinations of populations [7–11]. The degree of incompatibility between populations is also variable, and many intermediate strains are typically observed. These within species incompatibilities suggest that there are many intraspecific variations in mating signals and preferences. Those variations could either fix in isolated populations or become targets of sexual selection under some conditions and consequently result in divergent mating-associated characters among different populations. It is important to understand the precise features of such variations in signals and perceptions that could potentially lead to an incipient speciation.

Cuticular hydrocarbons are known to play an important role as a contact pheromone in *Drosophila* mate recognition (reviewed in [12, 13]). They are used to recognize conspecific mate [14–16], as well as to distinguish sex [17–19] and to evaluate the mating history of the females by males [20–22]. In *D. melanogaster*, many studies have shown that some female specific long chain hydrocarbons, especially C27 dienes, are attractive substances for males, and some of the male components, *cis*-vaccenyl acetate (cVA) and 7-tricosene (7:C23), are shown to reduce the female attractiveness [12, 13]. Furthermore, “pheromone-free females” were courted by males more rigorously than wild-type females [16, 23], which highlights the importance of both attractive and inhibitory components for the courtship induction [24].

The quantity of each cuticular hyrdrocarbon component varies among closely related species of *D. melanogaster* [25] and even within this species [26]. Also, the profile could be modified by different food conditions as shown in cactophilic *D. mojavensis* [27]. Therefore, making correct decisions based on variable contents of cuticular hydrocarbons may not be a trivial task. Thus, the differential sensitivity to different hydrocarbon components may evolve relatively fast and could be a source of variations for differential mate choice.

In this study, we analyze within-species variations in mate preference using *D. melanogaster*, whose mating-associated characters and their genetic bases are extensively studied. We particularly focus on one combination of conspecific strains that show low mating frequency, and investigate the role of cuticular hydrocarbon on this intraspecific mating incompatibility. Also, by comparing gas chromatograms of different strains, we attempt to identify candidate components in female cuticular hydrocarbon that have an inhibitory effect on mating.

## 2. Materials and Methods

### 2.1. *Drosophila* Strains

Five *D. melanogaster* inbred strains, 4 *D. simulans* inbred strains, and 3 *D. simulans* isofemale lines were used in this study. Mel6(G59), TW1(G23), KY02001(G20), BZ1(G23), and VAV1(G15) are wild-derived *D. melanogaster* inbred strains originated from Benin (West Africa), Taiwan, Japan, Brazil, and Tonga, respectively [30–32]. W86(G15), sim CH1(G65), sim 5(G69), and SEY4(G51) are wild-derived *D. simulans* inbred strains originated from Madagascar, USA., Congo, and Seycheleis Islands, respectively. Numbers in the parentheses indicate numbers of generations sibmated in the laboratory. S2 is a *D. simulans* isofemale line from Japan, which has been reported to be highly crossable to *D. melanogaster* [33]. Mel7 and Mel8 are *D. simulans* isofemale lines originated from Benin (West Africa). Flies were fed with regular sugar-yeast food and cultured at 23–25°C under a natural laboratory light condition.

### 2.2. No-Choice Design for Interspecific Mating Experiment

Interspecific no-choice mating experiment was conducted by placing females and males of different species together in a test vial. Unmated females and males were collected after lightly anesthetized with CO_2_ and were used 2 days after eclosion. Ten females from one species and 10 males from another species were put into a test vial (2.7 cm diameter × 10 cm height) filled with ~2.5 cm food at the bottom and capped with a sponge plug. The females were dissected after 24 hours to score the presence or absence of sperm in the spermatheca and/or seminal duct. Up to 2 occasional losses of samples were permitted per test vial. All the mating tests were done in the morning hours in a room temperature (~23°C) under a natural laboratory light condition.

### 2.3. Double-Choice Design

Double-choice mating experiment was conducted by placing females and males from 2 different strains together in a test vial. Unmated females and males were collected after lightly anesthetized with CO_2_ and were used 4 days after eclosion. Ten females and 5 males each from both strains were placed in a test vial (2.7 cm diameter × 10 cm height) filled with ~2.5 cm food at the bottom and capped with a sponge plug. The copulated pairs were aspirated out of the vial during the 3-hour test period and kept until the combinations of the mated strains were scored. Nine replicate test vials were scored for each experiment. The identification of strains was done using thoracic trident pigmentation intensity or abdominal pigmentation pattern. All the mating tests were done in the morning hours in a room temperature (~23°C) under a natural laboratory light condition.

### 2.4. No-Choice Design for Intraspecific Mating Experiment

Intraspecific no-choice mating experiment was conducted by placing one type of females and another type of males into a test vial. Unmated females and males were collected after lightly anesthetized with CO_2_ and were used 4 days after eclosion. Ten females and 10 males were placed in a test vial (2.7 cm diameter × 10 cm height) filled with ~2.5 cm food at the bottom and capped with a sponge plug. The number of copulated pairs was counted by aspirating them out of the vial during the 1-hour test period. Six replicate test vials were scored for each experiment. All the mating tests were done in the morning hours in a room temperature (~23°C) under a natural laboratory light condition.

### 2.5. Cuticular Hydrocarbon Transfer Experiment

Cuticular hydrocarbon transfer experiment was done by rub-off method as in Coyne et al. [14]. Cuticular hydrocarbon was transferred from TW1 to Mel6 by crowding 100 TW1 virgin females and 10 Mel6 virgin females in a limited space (2.7 cm diameter × 1 cm height) of a food vial for 4 days. Then the flies were lightly anesthetized with CO_2_ to select out Mel6 females. The thoracic trident pigmentation intensity was used to identify Mel6 females. The efficient transfer of cuticular hydrocarbon by this method was confirmed by gas chromatography (GC, data not shown). The control sets of females were obtained by crowding 110 Mel6 virgin females in the same limited space for the same period of time. Those females were used within 30 minutes after separated from the crowd.

### 2.6. Cuticular Hydrocarbon Analyses

Cuticular hydrocarbons were extracted from 4-day-old virgin females by n-hexane and analyzed by gas chromatography (GC). Cuticular hydrocarbons were extracted by washing 5 individuals in a glass culture tube with 500 *μ*L *n*-hexane for 5 min at room temperature. Then, *n*-hexane solution was transferred to a glass spits tube and stored in −20°C after the solvent was fully evaporated. Immediately before GC analyses, 100 *μ*L *n*-hexane was added to the tube, and 2 *μ*L of the resultant solution (equivalent to extract from ~0.1 individual) was used for the analyses. GC analyses were performed with a Shimazu GC-14A equipped with a DB-1 apolar column (length, 30 m; diameter, 0.25 mm; film thickness, 0.25 *μ*m; Agilent Technology Inc.) and flame ionization detector. Helium was used as the carrier gas. Injection was made in splitless mode for 1 minute at 300°C with a detector temperature of 300°C. The oven was programmed to hold at 80°C for 1 minute, and increased at 10°C/minute to 320°C, and held for 5 minutes.

## 3. Results

Premating isolation among *D. melanogaster* sibling species is not complete. For example, a certain level of mating occurs between *D. melanogaster* and *D. simulans* in the laboratory. If there are slight intraspecific differences in visual, acoustic, or chemical signals during the mating and differences in perception and response to those signals, they would show up as differential mating frequencies to a closely related species. In order to unveil these differences, we surveyed mating frequencies between different strains of *D. melanogaster* and *D. simulans*.

**Figure 1:**
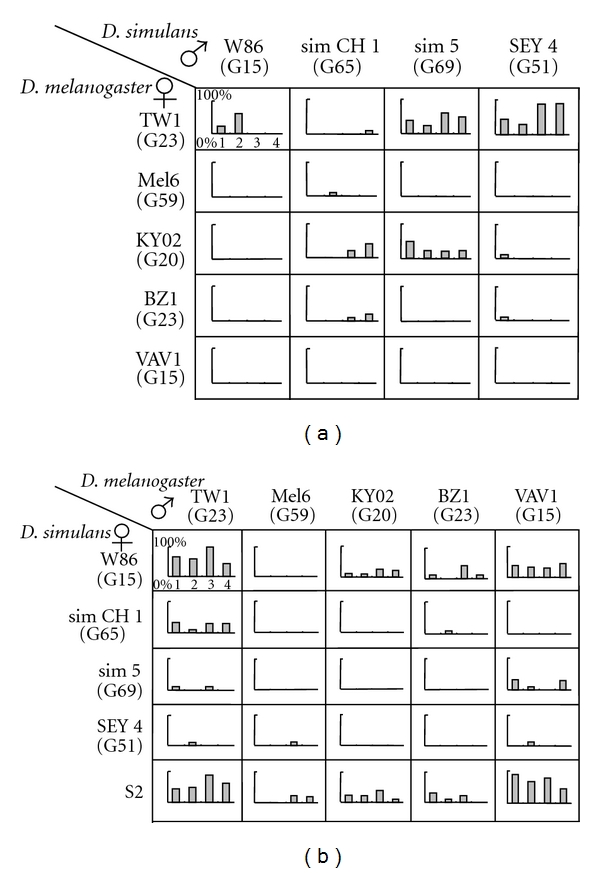
Mating frequency between strains from *D. melanogaster* and *D. simulans* by no-choice design. Vertical axis of each graph indicates % inseminated females after 24 hours. Each bar indicates result from a test vial. The first 2 replicate vials and the last 2 replicate vials were tested on a separate day. (a) Mating frequency between *D. melanogaster* females and *D. simulans* males. (b) Mating frequency between *D. simulans* females and *D. melanogaster* males.

The experiments were done by interspecific no-choice design, which involves placing 10 females and 10 males from different species into a test vial. The mating frequencies were assessed by scoring the number of inseminated females after 24 hours. For each cross, 2 vials were tested each day for 2 days, which gave 4 replicate data in total. Mating frequencies within 24 hours between the 2 species were not very high, but varied among strains (Figure 1).

Among those interspecific crosses, most notable differences were found between TW1 and Mel6. TW1 females readily mated with males from most of the *D. simulans* strains tested (Figure 1(a)), whereas females from Mel6 as well as those from VAV1 rarely mated with males from *D. simulans* strains. TW1 males also mated frequently with females from many of the *D. simulans* strains tested (Figure 1(b)), but Mel6 males seldom mated with *D. simulans* females. The mating patterns in most of other strains fell in between those two strains (Figures 1(a) and 1(b)). By the same experimental setting with 10 females and 10 males in each of the two test vials, 18 (90%) out of 20 Mel6 females mated with Mel6 males and 16 (84%) out of 19 TW1 females mated with TW1 males within 2 hours. The results indicated that individuals from Mel6 strain mated readily with individuals from their own strain but seemed to avoid mating with *D. simulans*.

Taken together, these results indicated that TW1 and Mel6 indeed have differential mating signals or responses to those signals or both. We also noticed during the experiments that Mel6 males rarely exhibited courtship behavior to *D. simulans* females when placed together in a same vial. This observation suggested that the male mate preference could be different between TW1 and Mel6.

**Figure 2:**
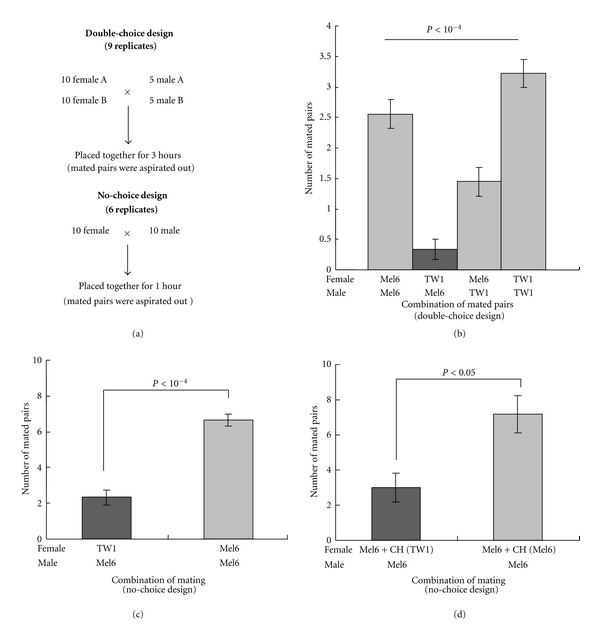
Mating experiment between TW1 and Mel6 by double-choice and no-choice design. (a) Description of the mating designs. (b) Number of mated pairs in the double-choice mating experiment. The pattern deviated from random mating (Cochran-Mantel-Haenszel exact test *P* < 10^−4^). (c) Number of mated pairs in the no-choice mating experiment between TW1 female and Mel6 male and that between Mel6 females and males. The difference between the two combinations of strains was significant (Student *t*-test, *t* = 7.27, df = 10, *P* < 10^−4^). (d) Number of mated pairs in the no-choice mating experiment between Mel6 males and Mel6 females coated by cuticular hydrocarbon of TW1 females, “Mel6 + CH(TW1)”, and that between Mel6 males and Mel6 females coated by cuticular hydrocarbon of their own, “Mel6 + CH (Mel6)”. The difference between the two combinations was significant (Student *t*-test, *t* = 3.08, df = 10, *P* = 0.012).

Since frequencies of successful mating with *D. simulans* differed between TW1 and Mel6, we suspected that the difference in mate preference may affect the mating between the two strains. In order to investigate whether the mating occurs randomly between the two strains, we first conducted mate choice experiment by double-choice design, which involves placing females and males from both strains together in a test vial (Figure 2(a)). As we suspected, the mating pattern deviated from random choice (Figure 2(b), Cochran-Mantel-Haenszel exact test (*P* < 10^−4^), and indicated that the mating between TW1 female and Mel6 male occurred at a very low frequency.

Together with the observation that Mel6 males rarely courted *D. simulans* females, we decided to focus our attention to mate preference by Mel6 male. First, the no-choice design was adopted, where only one type of females and one type of males were placed together in a mating vial (Figure 2(a)). The result showed that even by the no-choice experiment, the number of matings occurred within 1 hour between TW1 females and Mel6 males remained to be very small compared to that between Mel6 females and males (Figure 2(c)).

The low frequency mating between TW1 and Mel6 could be due to either male refusal or female rejection. In order to ask if there is Mel6 male refusal of TW1 female, we conducted the no-choice experiment by Mel6 female perfumed with cuticular hydrocarbons of TW1 female. Cuticular hydrocarbons can be transferred to other females by crowding the flies with the donor flies [14]. In order to see if cuticular hydrocarbons of TW1 have an inhibitory effect on mating, they were transferred to Mel6 females before conducting no-choice experiment. The experiment showed that Mel6 females coated with cuticular hydrocarbons from TW1 females mated less frequently with Mel6 males compared to the control Mel6 females (Figure 2(d)). This indicated that cuticular hydrocarbons of TW1 females have an inhibitory effect on Mel6 male mating behavior.

**Figure 3:**
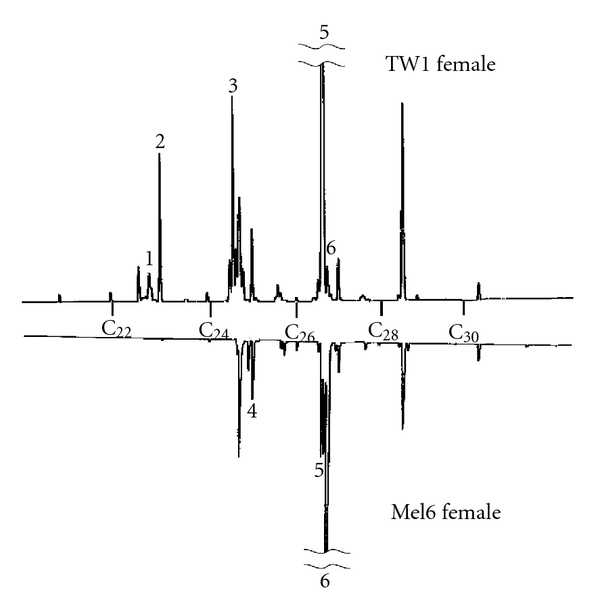
Mirrored gas chromatograms of *n*-hexane extracts of TW1 and Mel6 females. C_22_–C_30_ standard marker positions are indicated between the chromatograms. Each peak corresponds to hydrocarbon compound whose identity could be inferred from the previous studies [28, 29]. The peaks indicating qualitative differences (presence/absence or extreme high/low) between the two strains are marked by numbers. The inferred components for the peaks: peak #1: 7:C23; #2: C23; #3: 7,11:C25; #4: 5:C25; #5: 7,11:C27; #6: 5,9:C27 (+2-methylhexacosane).

We then compared the profiles of cuticular hydrocarbon components between the two strains by GC (Figure 3). The identities of the corresponding cuticular hydrocarbon components of the peaks were inferred from the previous studies [28, 29]. The peaks, which showed qualitative differences (presence/absence or extreme high/low) between the two strains, were marked by numbers in Figure 3. Their relative quantities (% area of the peaks) are shown in Table 1. Inseparable peaks and peaks with less than 2% of the whole peak area were not included in the comparison.

Mel6 females had a relatively simple content of cuticular hydrocarbons with almost no trace of peaks in the C23 group (around peaks #1 and #2), whereas the overall complexity of the TW1 female profile was similar to the typical wild type strains of *D. melanogaster* [12, 25]. The cuticular hydrocarbon profile of Mel6 females resembled that of Tai Y strain originated also from West Africa (Ivory Coast) with only traces of C23 hydrocarbons [12, 25].

The peaks #5 and #6 were inferred to be 7,11:C27 and 5,9:C27, respectively. However, 5,9:C27 peak is likely to be confounded with comigrating 2-methyl-hexacosane [24, 34]. The polymorphism in the ratio of these two C27 dienes has been well documented in the worldwide samples of *D. melanogaster* [24, 34, 35]. Although there is an association of *desat2* genotype, which is responsible for the polymorphism, with a sexual isolation between Zimbabwe and cosmopolitan populations of this species [36], this polymorphism does not seem to induce assortative mating pattern in general [34].

**Figure 4:**
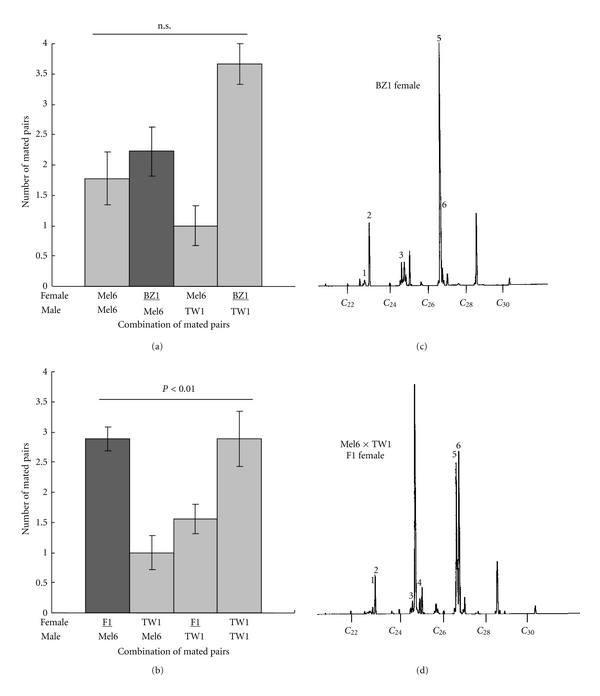
Mate discrimination by Mel6 males towards females with different cuticular hydrocarbon profiles. (a) Number of mated pairs in the double-choice mating experiment by substituting Mel6 females with BZ1 females in Figure 2(b). The pattern did not deviate from random mating (Cochran-Mantel-Haenszel exact test *P* > 0.10). (b) Number of mated pairs in the double-choice mating experiment by substituting TW1 females with F1 females (Mel6 × TW1) in Figure 2(b). The pattern deviated from random mating (Cochran-Mantel-Haenszel exact test *P* < 0.01). (c), (d) Gas chromatograms of *n*-hexane extracts of BZ1 females and F1 females (Mel6 × TW1), respectively. The numbers indicate corresponding peaks in Figure 3.

Next, we used two other types of females with different cuticular hydrocarbon profiles to test how mate preference of Mel6 males changes (Figure 4). Mel6 males did not discriminate between Mel6 and BZ1 females, which showed similar cuticular hydrocarbon profiles to TW1 females in peaks #1–6 except a smaller peak in peak #3 (Figures 4(a) and 4(c), Table 1). We also tested F1 females from Mel6 × TW1 cross, which also showed a smaller peak in peak #3 compared to that of TW1 female, and intermediate height peaks of #5 and #6 (Figures 4(b) and 4(d), Table 1). Mel6 males clearly favored these F1 females over TW1 females (Figure 4(b)), which gave a mating pattern similar to Figure 2(b). Previously known polymorphism in the ratio of 7,11:C27 (peak #5) and 5,9:C27 (peak #6) does not seem to affect the mate preference of Mel6 males. Taken together, these comparisons are consistent with the notion that the component in the TW1 female cuticular hydrocarbon, which has an inhibitory effect on mating with Mel6 males, is in the peak #3. The component of this peak is likely to be a C25 diene and is inferred to be 7,11:C25 from the previous literatures [28, 29].

**Figure 5:**
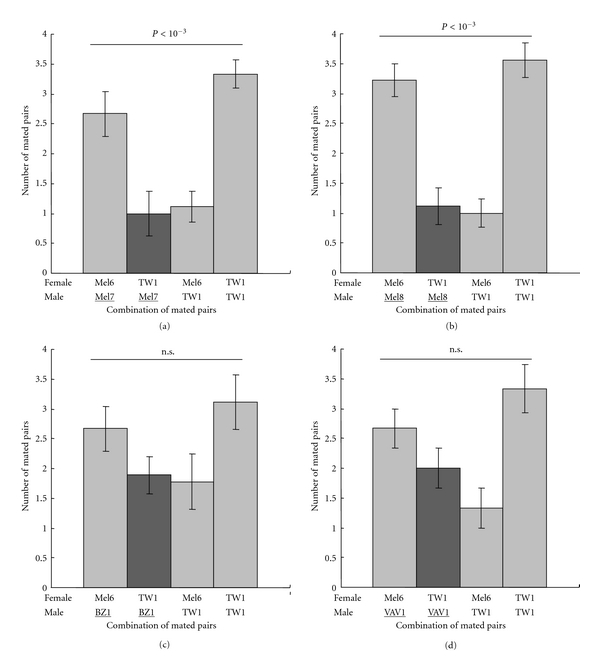
Mate discrimination by males from different strains. (a) Number of mated pairs in the double-choice mating experiment by substituting Mel6 males with Mel7 males in Figure 2(b). (b) Number of mated pairs in the double-choice mating experiment by substituting Mel6 males with Mel8 males in Figure 2(b). (c) Number of mated pairs in the double-choice mating experiment by substituting Mel6 males with BZ1 males in Figure 2(b). (d) Number of mated pairs in the double-choice mating experiment by substituting Mel6 males with VAV1 males in Figure 2(b). The substituted strain is indicated by an underline. The result of Cochran-Mantel-Haenszel exact test after figure-wide Bonferroni correction for multiple tests is shown for each experiment. Note that Mel7 and Mel8 strains are from the same collection site as Mel6.

In order to ask how general is this mate discrimination of Mel6 males against TW1 females, we tested 2 other strains originated from the same collection point of Mel6 in West Africa (Mel7 and Mel8) and strains from Brazil (BZ1) and Tonga (VAV1) to investigate whether their males show nonrandom mate choice between Mel6 and TW1 females. In order to avoid the effect of other preference factors, the male Mel6 was substituted by the test strain male in the double-choice experiment identical to Figures 2(a) and 2(b). The results in Figure 5 indicated that males from two other strains originated from the same collection site as Mel6 in West Africa mated more with Mel6 females than with TW1 females (Figures 5(a) and 5(b)) giving the similar pattern as in Figure 2(b), whereas strains from Brazil (BZ1) and Tonga (VAV1) did not show a clear refusal of mating with TW1 females (Figures 5(c) and 5(d)). These patterns suggest that Mel6-type mate discrimination is endemic to individuals from its collection site in West Africa.

## 4. Discussion

In order to analyze within species variations in mate preference, we first performed a survey of mating frequency between different strains of *D. melanogaster* and its sibling species, *D. simulans*. Crossability between these two species has been reported to be asymmetric; *D. melanogaster* females mate relatively easily with *D. simulans* males, but the reciprocal cross is more difficult [37, 38]. Our results did not follow this pattern (Figure 1). Not many *D. melanogaster* females mated with *D. simulans* males except those of TW1 (Figure 1(a)). In the reciprocal cross, in addition to S2 that has been documented as a highly crossable strain to *D. melanogaster* males [33], there was another strain W86 from Madagascar that mated well with *D. simulans* males (Figure 1(b)). Our results suggest that the asymmetry in crossability may depend highly on the strains tested.

What was more notable in our survey was that the crossability between the two sibling species was highly variable among strains (Figure 1), which indicated that the signals presented by either or both sexes and the perception and response to those signals are not completely fixed within each species. These intraspecific variations may have become more apparent in our survey using inbred strains (except S2), because some of the recessive alleles that affect those traits may have become homozygous during the inbreeding process. This inbreeding effect may have affected the asymmetry in interspecific crossability to some extent as well.

Because of the differential mating frequencies of TW1 and Mel6 against *D. simulans* strains (Figure 1), we predicted that their differences in courtship signals and perceptions may cause partial incompatibility between these strains. As predicted, low mating frequency between TW1 females and Mel6 males was observed (Figures 2(b) and 2(c)). Mel6 males were choosy in both interspecific and intraspecific crosses. The factors responsible for the low mating frequency between TW1 females and Mel6 males turned out to be a difference in cuticular hydrocarbon component and its perception. Since C25 dienes are not reported in the cuticular hydrocarbons of *D. simulans* females [12], different factors may be responsible for the interspecific mating frequency differences between these two strains. It is certainly of our interest to identify the factors involved in differential mate preferences against interspecific individuals.

There are several cases of partial incompatibility in mating between strains or populations of *D. melanogaster*. One is between populations from Zimbabwe (Z) and cosmopolitan (M) areas, in which case there is a strong behavior incompatibility between Z females and M males [7]. TW1 female-Mel6 male incompatibility is not part of this Z-M system, because the compatibility in the former is between a non-African female and an African male, which is opposite of the latter. Similarly, US and Caribbean populations exhibit partial incompatibility [10, 11] and there are also cases reported in Brazzaville, Congo [8] and at the “Evolution Canyon” in Israel [9] where two closely located populations show certain amount of premating isolation. To determine whether our observation of mating incompatibility between TW1 and Mel6 is another case of interpopulational sexual isolation or not, we should await a larger survey of strains, especially since we currently have found only one TW1 type strain. However, our study demonstrates that a single cue, a particular cuticular hydrocarbon blend, could produce differential responses of males from different strains within *D. melanogaster*.

The cuticular hydrocarbon transfer experiment showed that it is not the female rejection, but the male refusal of females, which brings about the reduced mating frequency between TW1 females and Mel6 males (Figure 2(d)). It is generally thought that females drive sexual isolation in *Drosophila*, because males are observed to court females largely indiscriminately while females seem to be able to effectively reject unpreferable males. However, our experiment clearly indicated that, in some cases, males actually do choose females according to the cuticular hydrocarbon profiles of females. This is not surprising since it is known that the female cuticular hydrocarbons contain components that exhibit both stimulatory and inhibitory effect on mating [16, 23, 24]. A known inhibitory sex pheromone for *D. melanogaster* males, 7-tricosene, is predominant in male cuticular hydrocarbon and perceived as a bitter stimulus for the males [19]. This molecule is at a normal quantity in TW1 females (peak #1, Table 1). Our interpretation that cuticular hydrocarbons of TW1 contain inhibitory factors instead of lacking stimulatory components comes from the fact that the only detectable peaks that had higher quantity in Mel6 were peaks #4 and #6 (Figure 3) and that both peaks were not present in BZ1 females that mated well with Mel6 males (Figure 4(a), Table 1). However, a subtle balance between the quantities of excitatory and inhibitory components may be critical for the decision making by males. Future assays using purified hydrocarbons should elucidate the combined effects of different components.

Under the assumption that cuticular hydrocarbon blend from TW1 female has inhibitory effect on mating in Mel6 males, we identified a candidate cuticular hydrocarbon component, a C25 diene, that has a deterrent effect on Mel6 males. The component is inferred to be 7,11:C25 from the previous literature [28, 29]. A large increase in 7,11:C25 (+13.2% of the total hydrocarbon amount) was reported as a consequence of an RNAi knockdown of an elongase gene, *eloF*, in *D. melanogaster* female [39]. Interestingly, those females showed decreased attractivity to Canton-S males. This observation is consistent with our conclusion that 7,11:C25 may serve as an inhibitory effect on the mate recognition of males. We should note that *eloF* knockdown females also showed marked decrease in 7,11:C29, which was not apparent in TW1 females (Figure 3).

Males from 3 strains originated from Benin (West Africa), Mel6, Mel7, and Mel8, did not readily mate with TW1 females, whereas 2 other strains from outside Africa showed no such discrimination (Figures 2 and 5). The cuticular hydrocarbon profiles of Mel6 (Figure 3), and another isofemale line from West Africa, Tai Y, were similar with only traces of C23 hydrocarbons [12, 25]. Females of Mel7 showed a similar profile to that of Mel6 as well (data not shown). These observations suggest that *D. melanogaster* in regions of West Africa may have different sensitivity or response to chemicals on the female cuticles. There are some identified olfactory and gustatory receptors mediating male courtship behavior in response to sex pheromones [40–44]. The differential expression of these receptors among males from different strains could be a cause of the differential mate choice.

Courtship behavior is affected by conditioning prior to courtship; unsuccessful courtship reduces subsequent courtship in males [45, 46]. It has been shown that flies can have memory of at least one cuticular hydrocarbon component, *cis*-vaccenyl acetate, during the conditioning [47]. In our experiments, males were kept in groups prior to the mating experiments, which may have affected the memories of unsuccessful male-male courtship. However, an elevated level of the C25 diene was not observed in Mel6 nor TW1 males (data not shown), which suggests that there was no differential learning associated with the candidate inhibitory factor.

Our study was the first case to suggest that a C25 diene produced by a wild-derived strain has an inhibitory effect on mating behavior of males and to show that the sensitivity to this chemical varies among strains. Such modification in sensitivity could become a potential isolation factor during incipient speciation. Further investigation of the precise mechanisms and genetics underlying these intraspecific variations may help entangle the evolution of complex intersexual communication and mating behavior in flies.

## Figures and Tables

**Table 1:** Relative quantity (% area) of the GC peaks designated in Figure 3. Sample sizes are shown under the strain names. Mean ± S.D. Values are shown.

Strain	Peak #1 7:C23^a^ (%)	Peak #2 C23^a^ (%)	Peak #3 7,11:C25^a^ (%)	Peak #4 5:C25^a^ (%)	Peak #5 7,11:C27^a^ (%)	Peak #6 5,9:C27^a^ (+2-methylhexacosane) (%)
TW1 (*n* = 3)	2.65 ± 0.07	6.44 ± 0.11	10.96 ± 1.25	0.00 ± 0.00	46.77 ± 0.61	2.22 ± 0.08
Mel6 (*n* = 3)	0.00 ± 0.00	0.00 ± 0.00	0.00 ± 0.00	2.67 ± 0.05	7.54 ± 6.53	58.77 ± 5.18
BZ1 (*n* = 2)	2.46 ± 0.31	8.64 ± 0.99	3.95 ± 0.07	0.00 ± 0.00	52.11 ± 0.33	3.06 ± 0.42
Mel6 × TW1 F1 (*n* = 2)	0.00 ± 0.00	4.43 ± 0.09	1.85 ± 0.02	1.98 ± 0.06	25.60 ± 0.34	19.55 ± 0.30

^a^Cuticular hydrocarbon components inferred from the previous studies [28, 29].

## References

[1] Greenspan RJ, Ferveur JF (2000). Courtship in *Drosophila*. *Annual Review of Genetics*.

[2] Coyne JA, Orr HA (1989). Patterns of speciation in *Drosophila*. *Evolution*.

[3] Coyne JA, Orr HA (1997). “Patterns of speciation in *Drosophila*” revisited. *Evolution*.

[4] Schug MD, Baines JF, Killon-Atwood A (2008). Evolution of mating isolation between populations of *Drosophila ananassae*. *Molecular Ecology*.

[5] Hirai Y, Kimura MT (1997). Incipient reproductive isolation between two morphs of *Drosophila elegans* (Diptera: Drosophilidae). *Biological Journal of the Linnean Society*.

[6] Ishii K, Hirai Y, Katagiri C, Kimura MT (2001). Sexual isolation and cuticular hydrocarbons in *Drosophila elegans*. *Heredity*.

[7] Wu CI, Hollocher H, Begun DJ, Aquadro CF, Xu Y, Wu ML (1995). Sexual isolation in *Drosophila melanogaster*: a possible case of incipient speciation. *Proceedings of the National Academy of Sciences of the United States of America*.

[8] Capy P, Veuille M, Paillette M, Jallon JM, Vouidibio J, David JR (2000). Sexual isolation of genetically differentiated sympatric populations of *Drosophila melanogaster* in Brazzaville, Congo: the first step towards speciation?. *Heredity*.

[9] Korol A, Rashkovetsky E, Iliadi K, Michalak P, Ronin Y, Nevo E (2000). Nonrandom mating in *Drosophila metanogaster* laboratory populations derived from closely adjacent ecologically contrasting slopes at “Evolution Canyon”. *Proceedings of the National Academy of Sciences of the United States of America*.

[10] Yukilevich R, True JR (2008). Incipient sexual isolation among cosmopolitan *Drosophila melanogaster* populations. *Evolution*.

[11] Yukilevich R, True JR (2008). African morphology, behavior and phermones underlie incipient sexual isolation between us and Caribbean *Drosophila melanogaster*. *Evolution*.

[12] Jallon JM (1984). A few chemical words exchanged by *Drosophila* during courtship and mating. *Behavior Genetics*.

[13] Ferveur JF (2005). Cuticular hydrocarbons: their evolution and roles in *Drosophila* pheromonal communication. *Behavior Genetics*.

[14] Coyne JA, Crittenden AP, Mah K (1994). Genetics of a pheromonal difference contributing to reproductive isolation in *Drosophila*. *Science*.

[15] Coyne JA, Oyama R (1995). Localization of pheromonal sexual dimorphism in *Drosophila melanogaster* and its effect on sexual isolation. *Proceedings of the National Academy of Sciences of the United States of America*.

[16] Savarit F, Sureau G, Cobb M, Ferveur JF (1999). Genetic elimination of known pheromones reveals the fundamental chemical bases of mating and isolation in *Drosophila*. *Proceedings of the National Academy of Sciences of the United States of America*.

[17] Antony C, Davis TL, Carlson DA, Pechine JM, Jallon JM (1985). Compared behavioral responses of male *Drosophila melanogaster* (Canton S) to natural and synthetic aphrodisiacs. *Journal of Chemical Ecology*.

[18] Grillet M, Dartevelle L, Ferveur JF (2006). A *Drosophila* male pheromone affects female sexual receptivity. *Proceedings of the Royal Society B: Biological Sciences*.

[19] Lacaille F, Hiroi M, Twele R (2007). An inhibitory sex pheromone tastes bitter for *Drosophila* males. *PLoS ONE*.

[20] Scott D (1986). Sexual mimicry regulates the attractiveness of mated *Drosophila melanogaster* females. *Proceedings of the National Academy of Sciences of the United States of America*.

[21] Scott D, Richmond RC, Carlson DA (1988). Pheromones exchanged during mating: a mechanism for mate assessment in *Drosophila*. *Animal Behaviour*.

[22] Scott D, Jackson LL (1990). The basis for control of post-mating sexual attractiveness by *Drosophila melanogaster* females. *Animal Behaviour*.

[23] Billeter JC, Atallah J, Krupp JJ, Millar JG, Levine JD (2009). Specialized cells tag sexual and species identity in *Drosophila melanogaster*. *Nature*.

[24] Ferveur JF, Sureau G (1996). Simultaneous influence on male courtship of stimulatory and inhibitory pheromones produced by live sex-mosaic *Drosophila melanogaster*. *Proceedings of the Royal Society B: Biological Sciences*.

[25] Jallon JM, David JR (1987). Variation in cuticular hydrocarbons among the eight species of the *Drosophila melanogaster* subgroup. *Evolution*.

[26] Ferveur JF, Cobb M, Boukella H, Jallon JM (1996). World-wide variation in *Drosophila melanogaster* sex pheromone: behavioural effects, genetic bases and potential evolutionary consequences. *Genetica*.

[27] Stennett MD, Etges WJ (1997). Premating isolation is determined by larval rearing substrates in cactophilic *Drosophila mojavensis*. III. Epicuticular hydrocarbon variation is determined by use of different host plants in *Drosophila mojavensis* and *Drosophila arizonae*. *Journal of Chemical Ecology*.

[28] Marcillac F, Bousquet F, Alabouvette J, Savarit F, Ferveur JF (2005). A mutation with major effects on *Drosophila melanogaster* sex pheromones. *Genetics*.

[29] Foley B, Chenoweth SF, Nuzhdin SV, Blows MW (2007). Natural genetic variation in cuticular hydrocarbon expression in male and female *Drosophila melanogaster*. *Genetics*.

[30] Takahashi A, Takano-Shimizu T (2005). A high-frequency null mutant of an odorant-binding protein gene, *Obp57e*, in *Drosophila melanogaster*. *Genetics*.

[31] Takahashi A, Takahashi K, Ueda R, Takano-Shimizu T (2007). Natural variation of *ebony* gene controlling thoracic pigmentation in *Drosophila melanogaster*. *Genetics*.

[32] Fujikawa K, Takahashi A, Nishimura A, Itoh M, Takano-Shimizu T, Ozaki M (2009). Characteristics of genes up-regulated and down-regulated after 24 h starvation in the head of *Drosophila*. *Gene*.

[33] Uenoyama T, Inoue Y (1995). Genetic studies on premating isolation in *Drosophila simulans*. I. A D. simulans line highly crossable with *D. melanogaster*. *Japanese Journal of Genetics*.

[34] Coyne JA, Wicker-Thomas C, Jallon JM (1999). A gene responsible for a cuticular hydrocarbon polymorphism in *Drosophila melanogaster*. *Genetical Research*.

[35] Takahashi A, Tsaur SC, Coyne JA, Wu CI (2001). The nucleotide changes governing cuticular hydrocarbon variation and their evolution in *Drosophila melanogaster*. *Proceedings of the National Academy of Sciences of the United States of America*.

[36] Fang S, Takahashi A, Wu CI (2002). A mutation in the promoter of *desaturase 2* is correlated with sexual isolation between *Drosophila* behavioral races. *Genetics*.

[37] Das A, Mohanty S, Capy P, David JR (1995). Mating propensity of Indian *Drosophila melanogaster* populations with *D.simulans*: a nonadaptive latitudinal cline. *Heredity*.

[38] Moulin B, Aubin T, Jallon JM (2004). Why there is a one-way crossability between *D. melanogaster* and *D. simulans*?. *Genetica*.

[39] Chertemps T, Duportets L, Labeur C (2007). A female-biased expressed elongase involved in long-chain hydrocarbon biosynthesis and courtship behavior in *Drosophila melanogaster*. *Proceedings of the National Academy of Sciences of the United States of America*.

[40] Bray S, Amrein H (2003). A putative *Drosophila* pheromone receptor expressed in male-specific taste neurons is required for efficient courtship. *Neuron*.

[41] Kurtovic A, Widmer A, Dickson BJ (2007). A single class of olfactory neurons mediates behavioural responses to a *Drosophila* sex pheromone. *Nature*.

[42] van Naters WVDG, Carlson JR (2007). Receptors and neurons for fly odors in *Drosophila*. *Current Biology*.

[43] Moon SJ, Lee Y, Jiao Y, Montell C (2009). A *Drosophila* gustatory receptor essential for aversive taste and inhibiting male-to-male courtship. *Current Biology*.

[44] Wang L, Han X, Mehren J (2011). Hierarchical chemosensory regulation of male-male social interactions in *Drosophila*. *Nature Neuroscience*.

[45] Siegel RW, Hall JC (1979). Conditioned responses in courtship behavior of normal and mutant *Drosophila*. *Proceedings of the National Academy of Sciences of the United States of America*.

[46] Ejima A, Smith BPC, Lucas C, Levine JD, Griffith LC (2005). Sequential learning of pheromonal cues modulates memory consolidation in trainer-specific associative courtship conditioning. *Current Biology*.

[47] Ejima A, Smith BPC, Lucas C (2007). Generalization of courtship learning in *Drosophila* is mediated by *cis*- vaccenyl acetate. *Current Biology*.

